# Metal-organic frameworks-mediated reactive oxygen species modulation in the tumor microenvironment of digestive system malignancies: latest advances

**DOI:** 10.3389/fchem.2026.1809650

**Published:** 2026-04-15

**Authors:** Wei Liu, Chengxin Liu, Meng Wu, Siwen Liu

**Affiliations:** Department of Radiology, The Second Hospital of Jilin University, Changchun, China

**Keywords:** digestive system, malignancies, MOFs, nanomaterials, ROS, therapy, TME

## Abstract

Digestive system malignancies, such as gastric cancer (GC), colorectal cancer (CRC), and hepatocellular carcinoma (HCC), have become a major global challenge in cancer therapy. These tumors are characterized by high morbidity, strong metastatic potential, and significant chemoresistance, thus posing a severe threat to human digestive health. One of the primary mechanisms underlying the malignant progression of tumors and the development of drug-resistant phenotypes is the imbalance of redox homeostasis in the tumor microenvironment (TME). Its dysregulation is strongly associated with the increased proliferation and invasive potential of tumor cells. As key regulatory molecules in the redox network of the TME, reactive oxygen species (ROS) render precise modulation of their levels a promising strategy for cancer therapy. This approach can not only effectively induce programmed cell death in tumor cells but also reverse the immunosuppressive state of the TME, thus offering novel therapeutic targets for anti-tumor treatment. Metal-organic frameworks (MOFs) are closely associated with metal-based nanomaterials, acting as their excellent precursors and enabling precise modulation of their structures and properties through hybridization. The demand for the precise regulation of ROS in the TME of digestive system malignancies is perfectly met by MOFs, which have emerged as a highly promising nanoplatform for this research field. MOFs have unique advantages such as precisely designable architectures, abundant metallic catalytic active sites, TME-responsive degradation, and high drug loading capacity. This review elucidates the mechanisms underlying MOF-mediated ROS modulation in the TME and highlights its applications in various digestive system malignancies.

## 1 Introduction

Gastric cancer (GC), colorectal cancer (CRC), hepatocellular carcinoma (HCC), pancreatic cancer (PC), esophageal cancer (EC), and other cancers of the digestive tract have poor prognoses and high incidence rates. They represent a significant burden on the global public health system and are ranked among the most common malignancies globally in terms of both incidence and death (Danpanichkul et al., 2024; Morgan et al., 2023). Currently, surgical resection, radiotherapy, chemotherapy, and immunotherapy dominate the clinical therapeutic modalities for such cancers; however, these approaches provide limited therapeutic success and are confronted with considerable clinical bottlenecks. Because of the occult nature of early-stage tumors and residual micrometastases after surgery, surgical resection is prone to disease recurrence (Jeong et al., 2026). Chemotherapy and radiation therapy frequently have reduced clinical efficacy due to multidrug resistance in tumor cells (Valenzuela et al., 2025; Xu et al., 2025); immunotherapy, on the other hand, is unable to achieve its best therapeutic effects because of the intrinsic immunosuppressive tumor microenvironment (TME) (Luo et al., 2025).

The malignant phenotypes of tumors are intimately associated with the unique redox imbalance features present in the TME of digestive system malignancies. To maintain a dynamic homeostasis of high reactive oxygen species (ROS) production and high antioxidant capacity to adapt to the demands of malignant proliferation, tumor cells activate endogenous oxidase systems, which in turn sustain a high level of ROS production. At the same time, they upregulate the expression of antioxidant substances such as glutathione (GSH) and nicotinamide adenine dinucleotide phosphate (NADPH) (Kračun et al., 2025). Moreover, excessive ROS leads to functional exhaustion of immune cells such as T lymphocytes and macrophages, creating a redox-dependent immunosuppressive milieu that promotes tumor growth and chemoresistance (Shah et al., 2024). Therefore, upsetting TME redox equilibrium and precisely modulating ROS have become significant breakthroughs and key therapeutic targets for the treatment of cancers of the digestive system.

Small-molecule ROS inducers and antioxidant inhibitors are two examples of conventional ROS-modulating strategies that are universally beset by inherent problems, such as poor targeting ability, low ROS generation efficiency, and short action duration. In addition, these strategies often cause oxidative stress damage to normal tissues, which significantly hinders their clinical translation and practical application (Glorieux et al., 2024; Xu et al., 2024). However, as a family of crystalline porous materials constructed from metal ions/clusters and organic ligands via coordinate bonds, metal-organic frameworks (MOFs) feature exceptional qualities comprising excellent structural tunability, numerous metallic catalytic active sites, and tunable physicochemical properties (Liu et al., 2025; Soman et al., 2025). MOFs generate ROS, including hydroxyl radicals and superoxide anions, through Fenton-like reactions at metal centers and electron transfer of organic ligands under external stimulation. MOFs make it possible to logically construct pore size, catalytic activity, and surface features in a directed manner by carefully choosing metal nodes, including Fe^3+^, Cu^2+^, and Mn^2+^, and organic ligands, like carboxylic acids and imidazoles (Yu et al., 2025). These materials undergo regulated disintegration in response to TME-specific microenvironmental signals, including acidic pH, high GSH concentration, and enzymatic hydrolysis conditions, leading to tumor-targeted ROS regulation and abrogating off-target oxidative injury to normal tissues. Furthermore, MOFs’ ultra-large specific surface area allows them to efficiently load a variety of therapeutic agents, realizing the synergistic enhancement of oxidative therapy with other treatment modalities (Tian et al., 2021). In contrast to conventional porous materials, MOFs allow for atomic-level structural and functional control and are easily functionalized for stimuli-responsive release, drug delivery, and catalysis. MOFs have significant advantages in TME manipulation because of these unique chemical characteristics.

**FIGURE 1:**
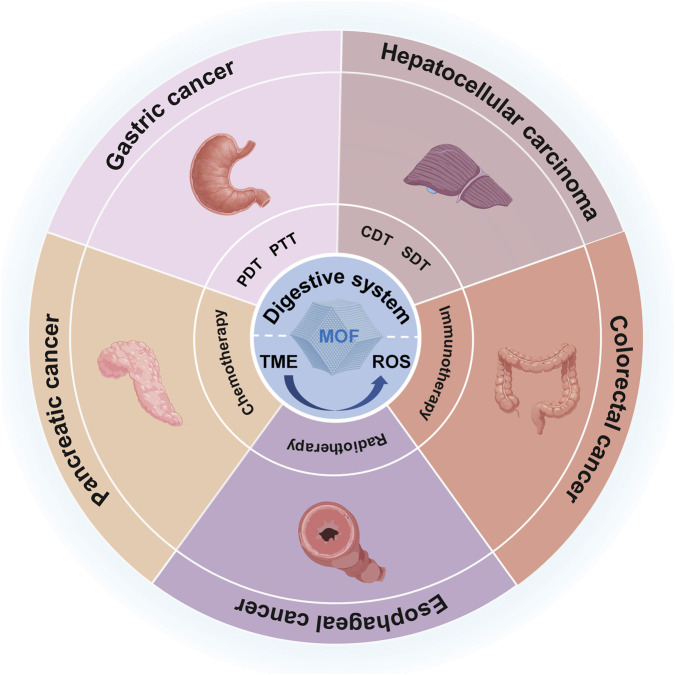
MOF-mediated ROS modulation in the TME for the diagnosis and therapy of digestive system Malignancies.

This review examines the fundamental mechanisms of MOF-mediated ROS modulation in the TME, details pertinent research advancements and application contexts of MOFs for the targeted modulation of ROS in relation to various digestive system malignancies, and additionally investigates the challenges and future directions in this domain (Figure 1).

## 2 Core mechanisms of MOF-mediated ROS modulation in the TME

The principal regulatory effects of MOFs for ROS in TME are predominantly realized through three specific mechanisms: generation of catalytic ROS (Cao et al., 2022), reduction of antioxidant compounds (Cheng et al., 2021; Fu et al., 2021), and targeted delivery of ROS-associated drugs (Lei et al., 2018; Zhou et al., 2021). Significantly, these three pathways operate synergistically rather than autonomously (Yang et al., 2025).

### 2.1 Generation of catalytic ROS

In the distinctive porous crystalline structures of MOFs, metallic ions establish extremely effective catalytic active sites by precise coordination with organic ligands. These sites can precisely replicate the catalytic activity of endogenous oxidoreductases (e.g., peroxidases, superoxide dismutases) and skillfully adjust to the tumor-specific microenvironmental signals of the digestive system malignancy TME, including acidic pH, hypoxia, and elevated reduction potential (Bu et al., 2023; Liu et al., 2022a; Yayun et al., 2025). Activated by tumor-specific microenvironments, MOFs can effectively catalyze endogenous substrates (e.g., H_2_O_2_) within tumor tissues to initiate Fenton or Fenton-like redox reactions, which continuously produce highly cytotoxic ROS (·OH, O_2_·^-^) and progressively disturb the intrinsic dynamic ROS balance upheld by tumor cells (Gao et al., 2023; Koo et al., 2022). Excessive accumulation of ROS causes significant oxidative stress damage in tumor cells, disrupts the normal structure and biological function of intracellular DNA, proteins, and lipids, and ultimately facilitates efficient *in situ* ROS accumulation within the local TME, thereby establishing a robust foundation for subsequent antitumor effects (An et al., 2024).

### 2.2 Reduction of antioxidant compounds

MOFs can specifically deplete intracellular antioxidant substances in tumor cells via rational structural design, thereby eradicating their ROS-scavenging capacity at the source. On the one hand, metallic ions in MOFs can initiate specific chelation reactions with highly expressed GSH in the TME, which proceed with an irreversible nature and exhaust intracellular GSH reserves with high efficiency, directly disrupting the intracellular reducing microenvironment (Chen et al., 2023a; Wang et al., 2020). Additionally, MOFs can promote oxidative reactions via their inherent catalytic properties to selectively decompose vital antioxidant coenzymes like NADPH, which is crucial for maintaining the function of antioxidant enzymes and the regeneration of GSH (Li et al., 2023). This dual approach jointly establishes a conducive local TME for the following extensive accumulation of ROS.

### 2.3 Targeted delivery of ROS-associated drugs

Characterized by unique advantages such as a high specific surface area, adjustable pore size, and ease of surface functionalization, MOFs function as optimal nanocarriers for the effective loading and targeted delivery of ROS-related pharmaceuticals. Their extensive specific surface area enhances drug loading capacity, while the adjustable pore size facilitates the selective encapsulation of therapeutic agents with differing molecular weights, like the chemotherapeutic agent doxorubicin (Ibrahim et al., 2022), porphyrin-based photosensitizers (Shano et al., 2024), and ROS inducers (Al Sharabati et al., 2022), thereby minimizing premature drug leakage. Modifying the surfaces of MOFs with targeting moieties like folic acid (Cai et al., 2020) and transferrin (Chai et al., 2025) enables the accurate identification of overexpressed specific receptors on the surfaces of malignant cells in the digestive system, resulting in targeted accumulation of therapeutic agents at tumor sites and reducing toxic side effects on normal tissues.

The intrinsic structure and composition of MOFs are directly linked to their capacity to control ROS in the TME via these three routes. Through Fenton-like reactions in the TME, the metal nodes in MOFs can function as catalytically active centers and directly produce ROS. To efficiently deplete antioxidants like GSH and upset the intracellular redox balance, the framework can be functionally altered. Additionally, MOFs’ enormous specific surface areas and adjustable pore architectures allow for effective loading and targeted delivery of medications linked to ROS. Thus, MOFs’ comprehensive modulation of ROS in the TME is supported by their exceptional drug-loading capacity, unique catalytic characteristics, and structural designability.

## 3 Application of MOFs in ROS modulation within the TME of digestive system malignancies

Recently, with the steady advancement and innovation of nanotechnology and the in-depth exploration of pathogenic mechanisms underlying digestive system malignancies, MOFs have found increasingly extensive applications in the digestive system field and notably exhibited their distinctive advantages, especially in tumor diagnosis and therapy (Table 1).

**TABLE 1:** Clinical applications of different MOFs for digestive system malignancies.

Nanoplatform	Metal node	Therapeutic method	Tumor type	References
Oxa@MIL-100 (Fe)	Fe	Chemodynamic Therapy	Gastric cancer	Sun et al. (2024a)
ZTC@M	Zn	Sonodynamic Therapy	Gastric cancer	Yu et al. (2022)
PCN-222/Au/DOX	Zr	Photodynamic Therapy	Gastric cancer	Tehrani Nejad et al. (2025)
Mn-MOF	Mn	Sonodynamic Therapy	Hepatocellular carcinoma	Xu et al. (2021)
Sor@Fe-MOF	Fe	Chemotherapy	Hepatocellular carcinoma	Yan et al. (2025)
AE-FeMn/FA	Fe, Mn	Immunotherapy	Hepatocellular carcinoma	Xie et al. (2025)
Fe-MOF/Cap	Fe	Immunotherapy	Hepatocellular carcinoma	Zhao et al. (2025)
GMTF	Mn	Chemodynamic Therapy, Sonodynamic Therapy	Colorectal cancer	Hang et al. (2024)
Bio-MOF	Fe	Chemotherapy	Colorectal cancer	Babaei et al. (2025)
OIMH NPs	Fe	Photothermal Therapy	Colorectal cancer	Liu et al. (2022b)
Cu-Olsa nanoMOF	Cu	Chemodynamic Therapy	Colorectal cancer	Li et al. (2022a)
ultra-small Ti-TCPP MOF	Ti	Sonodynamic Therapy	Pancreatic cancer	Zhang et al. (2021)
HMC	Zn	Sonodynamic Therapy	Pancreatic cancer	Chen et al. (2025)
CDCZA (Cur/CDDP/Cu/ZIF8@Au)	Zn	Chemodynamic Therapy	Esophageal cancer	Sun et al. (2024b)
UiO-66-NH_2_(Hf)	Hf	Radiotherapy	Esophageal cancer	Zhou et al. (2022)

### 3.1 GC

MOFs have been engineered into various functionalized formulations for ROS modulation within the TME of GC (Mirzanejad et al., 2025; Wang et al., 2025). Sun et al. (2024a) developed the Oxa@MIL-100(Fe) nanoplatform by incorporating oxaliplatin onto the iron-based MIL-100(Fe) nanocarrier. The main mechanism relies on ROS-mediated synergy between Fenton reaction-induced ferroptosis and chemotherapy-triggered apoptosis. MIL-100(Fe) dissociates in the acidic TME of GC to release Fe^3+^ and oxaliplatin; Fe^3+^ is reduced to Fe^2+^ by GSH, which triggers the Fenton reaction for •OH generation. This redox cycle depletes intracellular GSH and inhibits GPX4, thereby inducing ferroptosis. Oxaliplatin induces apoptosis in tumor cells by damaging cellular DNA, while the porous structure of MOFs facilitates the simultaneous release of Fe^3+^ and oxaliplatin. This synchronous release not only mitigates the toxic side effects of oxaliplatin but also amplifies oxidative damage via ROS to enhance tumoricidal efficiency, thus exerting a potent cytotoxic effect against GC cells. The combination of these two mechanisms substantially improves the therapeutic efficacy for GC. Furthermore, ZIF-8 was synthesized to co-encapsulate Chlorin e6 (Ce6) and tirapazamine (TPZ) due to its superior encapsulation capabilities. Subsequently, a biomimetic nanoplatform (ZTC@M) was developed by modifying the surface of ZIF-8 with GC cell membranes, thereby integrating sonodynamic and chemotherapeutic functionalities. The combined effects of these two therapeutic modalities effectively cause pyroptosis in GC cells and have a significant antitumor effect, hence offering a promising new strategy for the clinical treatment of GC (Yu et al., 2022). *Helicobacter pylori* (*H. pylori*) is a well-recognized risk factor for GC development. A ternary nanocomposite PCN-222/Au/DOX with dual functions of anti-GC activity and *H. pylori* photodynamic antibacterial activity was successfully prepared in a recent study. For GC cells, this nanocomposite generates ROS via the photodynamic effect to induce cellular apoptosis and exerts a remarkable tumor-suppressive effect through the synergistic enhancement of photodynamic therapy with chemotherapy. For *H. pylori* infection, a prevalent comorbidity of GC, the nanocomposite is utilized to disrupt bacterial cellular structures through the photodynamic effect when exposed to light, resulting in a significant photobactericidal effect. This study presents an innovative nanomaterial-based approach for the integrated diagnosis and treatment of GC associated with *H. pylori* co-infection (Tehrani Nejad et al., 2025).

### 3.2 HCC

The TME of HCC is distinguished by hallmark characteristics of hypoxia, immunosuppression, and disrupted oxidative metabolism. The modulation of ROS has become a pivotal strategy for improving this malignant microenvironment and enhancing the therapeutic efficacy of HCC (Bao and Wong, 2021; Liang et al., 2025). Xu et al. (2021) synthesized a manganese porphyrin-based MOF (Mn-MOF), in which the catalase-like activity of manganese ions enabled the decomposition of H_2_O_2_ in the TME of HCC to generate oxygen. This process effectively relieves hypoxia in HCC tissues and provides favorable conditions for ROS generation. Moreover, ultrasonic irradiation can induce the porphyrin ligands inside the material to produce ROS, while manganese ions accelerate the cycling of iron ions to enhance the Fenton reaction. The massive accumulation of ROS depleted intracellular GSH, ultimately inducing ferroptosis in HCC cells through a synergistic effect. This nanoplatform realized the synergistic therapy of sonodynamic therapy (SDT) and ferroptosis induction for hypoxic HCC, exerting a remarkable inhibitory effect on xenograft tumors with no obvious hepatic or renal toxicity, thus offering a novel avenue for HCC treatment. In addition, an Fe (III)-based MOF nanocarrier was constructed to encapsulate sorafenib (Sor), forming the Sor@Fe-MOF nanoplatform. Iron ions released from this platform catalyzed the conversion of H_2_O_2_ in the TME of HCC to generate ROS, while Sor modulated ferroptosis-related signaling pathways; the two components acted in synergy to enhance ferroptosis in HCC cells. Meanwhile, via ROS modulation, this material effectively remodeled the immune TME of HCC, increased the infiltration of cytotoxic T lymphocytes (CTLs) in tumor tissues, promoted the activation and tumor penetration of immune cells, and synergistically enhanced the antitumor immune response. This approach not only overcame chemoresistance but also substantially improved the therapeutic efficacy of HCC(43). Xie et al. (2025) modified FeMn-MOF nanomaterials with folic acid (FA) and effectively encapsulated aloe-emodin (AE) because of their porous structures, ultimately creating the AE-FeMn/FA nanoplatform. This platform achieved active targeting of HCC cells and responsive and precise drug release in response to the acidic microenvironment and elevated H_2_O_2_ levels characteristic of the TME. Simultaneously, the Fe-Mn bimetallic skeleton of the material exhibited Fenton-like catalytic activity, enabling massive ROS generation, and acted in synergy with AE to induce pyroptosis in HCC cells for enhancing the antitumor immune response. The synergistic improvement of immunotherapy and chemotherapy for HCC was achieved through the regulation of ROS. Post-incomplete radiofrequency ablation (iRFA) of HCC is associated with an immunosuppressive TME, disrupted oxidative equilibrium, and a significantly elevated risk of metastasis, which continue to pose substantial clinical challenges. Zhao et al. (2025) synthesized the capmatinib (Cap)-loaded Fe-MOF/Cap nanoplatform, which achieved the efficient loading and targeted delivery of Cap via the porous structure of Fe-MOF, thus providing a novel and effective strategy for the adjuvant therapy of HCC after ablation.

### 3.3 CRC

The TME of CRC is marked by a dense extracellular matrix (ECM), inadequate tumor infiltration, and variable local hydrogen peroxide concentrations (Gong et al., 2025; Zhang et al., 2024). Recent studies have predominantly concentrated on the development of composite MOF systems to augment ROS modulation efficiency and increase tumor penetrability. Hang et al., (2024) successfully created metal-organic framework nanosheets (GMTF) that specifically target colon cancer and disintegrate in its acidic TME, affording synergistic chemodynamic therapy (CDT) and SDT while including magnetic resonance imaging (MRI) functionality. Significantly, when combined with ultrasonic irradiation, it demonstrates a pronounced inhibitory impact on colon cancer, efficiently curtails hepatic metastasis, and exhibits no discernible harm to normal tissues. A multifunctional biodegradable iron-based MOF (bio-MOF) incorporated with curcumin was developed in a separate investigation. Following precise modification, this material can be selectively concentrated in colorectal tumor tissues; it decomposes in reaction to the acidic TME, liberating curcumin and iron ions to provoke oxidative stress through ROS generation, and collaboratively influences pertinent signaling pathways to initiate cancer cell apoptosis. It additionally incorporates imaging capabilities to facilitate theranostics. This system significantly inhibits CRC growth, with its therapeutic efficacy markedly superior to that of free curcumin. Furthermore, it has superior biocompatibility, does not significantly disrupt the normal intestinal microbiota, and hence offers a fresh perspective for the targeted treatment of CRC (49). Liu et al. (2022b) effectively developed the OIMH NPs nanotherapeutic platform, which can be selectively concentrated in colorectal tumor tissues and incorporates photoacoustic imaging capabilities for real-time visual guidance throughout the therapeutic procedure. Under near-infrared laser irradiation, it facilitates the combination of chemotherapy and photothermal therapy (PTT), promotes immunogenic cell death (ICD) in tumor cells by substantial ROS accumulation, and improves the immunosuppressive TME to augment the effectiveness of immunotherapy. This strategy significantly inhibits CRC, effectively diminishes the harmful side effects of monotherapy, and so offers a novel strategy for the comprehensive treatment of CRC. Additionally, a Cu-Olsa nanoMOF was produced in a separate work, which combines catalytic and epigenetic regulatory capabilities. It can stimulate ROS production in the TME of CRC to provoke oxidative stress while concurrently augmenting the anticancer effect through epigenetic modulation. Possessing exceptional targeting capabilities, it markedly suppresses the proliferation and spread of CRC while exhibiting minimal damage to normal cells, thus presenting a novel approach for CRC treatment (Li et al., 2022a).

### 3.4 PC and EC

Currently, investigations on MOF-mediated ROS modulation within the TME of PC and EC are still very scarce. An innovative ultra-small Ti-TCPP MOF with inherent nuclear-targeting ability has been developed; when stimulated by low-intensity ultrasound, it induces sonodynamic effects, specifically targets the nuclei of PC cells, and produces ROS with great efficacy. This nanomaterial addresses the hypoxic constraints of traditional SDT, therefore providing an innovative nuclear-targeting ROS-enhanced therapeutic approach for PC (Zhang et al., 2021). Chen et al. (2025) synthesized hollow mesoporous carbon (HMC) nanoparticles that incorporate SDT and immunomodulation capabilities. This material, due to its intrinsic porphyrin-like structure, may efficiently create ROS under ultrasonic irradiation, effectively eliminating PC cells and triggering their ICD. When used in conjunction with a PD-L1 inhibitor, it effectively undermines the immunosuppressive TME of PC and amplifies the host’s antitumor immune response. This system demonstrates significant tumor-suppressive efficacy with excellent biocompatibility and functions as a versatile drug carrier suitable for various combination therapeutic strategies, thereby offering a novel approach for sonodynamic-immunotherapeutic synergy in the treatment of PC. In addition, Sun et al. (2024b) created the CDCZA (Cur/CDDP/Cu/ZIF8@Au) nanoplatform, which promotes ROS modulation and chemosensitization in the esophageal cancer TME. This method significantly suppressed EC proliferation in both *in vitro* and *in vivo* models, substantially diminished cisplatin resistance in cancer cells, and alleviated the systemic toxic side effects of chemotherapy, thereby offering a unique approach for the chemosensitization of EC. A UiO-66-NH_2_(Hf) nanoplatform has been developed; a hafnium-based metal-organic framework was produced utilizing hafnium ions as the metal source and amino terephthalic acid as the organic ligand, serving as a radiosensitizer for EC (Zhou et al., 2022). The elevated atomic number of hafnium boosts X-ray absorption and promotes ROS formation during radiation, resulting in DNA damage in tumor cells and initiating apoptosis, hence augmenting the efficacy of radiotherapy. Both *in vitro* and *in vivo* studies have confirmed its significant inhibitory effect on EC; furthermore, this nanoparticle demonstrates exceptional stability in physiological conditions and advantageous biocompatibility, presenting a novel direction for radiosensitization of EC through ROS modulation.

The TME of various cancers of the digestive tract varies significantly from one another. For example, HCC involves hepatic clearance and Kupffer cell-mediated uptake; CRC is characterized by microbiota-related effects and a thick mucus barrier; PC presents dense stromal fibrosis and insufficient perfusion; and GC typically displays an acidic microenvironment and distinct mucosal barrier characteristics. The stability, tumor penetration, and ROS-mediated therapeutic efficacy of MOFs can all be strongly impacted by these subtype-specific tumor microenvironmental characteristics.

## 4 Conclusion

Low early diagnosis rates, high invasion and metastatic potential, highly immunosuppressive TME, and frequent multidrug resistance are the main reasons why digestive system cancers are difficult to cure. Furthermore, monotherapy can scarcely cover all malignant phenotypes due to the significant heterogeneity of tumors, which leads to consistently high recurrence rates. MOFs possess adjustable pores and plentiful metallic active sites, displaying excellent efficacy in ROS modulation within the TME of digestive system cancers. They enable drug loading and regulated release, *in situ* ROS generation for tumor suppression, and theranostic integration, enabling fresh techniques for tumor therapy. However, various barriers complicate the clinical translation of MOFs. Their structural integrity and impacts on the gut microbiota await long-term safety validation (Chen et al., 2021; Simon-Yarza et al., 2016); insufficient endogenous H_2_O_2_, tumor heterogeneity, and other issues in advanced tumors compromise therapeutic efficacy (Li et al., 2022b), alongside delivery challenges including poor tumor penetration, oral degradability, and inefficient deep-tissue delivery (Raza and Wu, 2024). The delivery issues are intricately linked to the swift clearance by the reticuloendothelial system (RES) and substantial buildup in the liver and spleen following intravenous treatment, with inadequate tumor penetration and strong ECM barriers in PC. Advanced design strategies, like size/surface engineering, biomimetic coatings, ECM manipulation, and active targeting, may successfully mitigate these challenges and enhance efficient and targeted administration *in vivo* (Chen et al., 2023b). In addition, chemotherapy, radiation, targeted therapy, and immunotherapy are frequently employed in addition to ROS-mediated oxidative damage therapy. These treatments can effectively destroy tumors, reverse drug resistance, limit proliferation, and boost immunity. In the future, MOFs are expected to pioneer the precise theranostics of digestive system cancers.
